# Cost-effectiveness analysis of paediatric mental health interventions: a systematic review of model-based economic evaluations

**DOI:** 10.1186/s12913-022-07939-x

**Published:** 2022-04-22

**Authors:** Sanjeewa Kularatna, Ruvini Hettiarachchi, Sameera Senanayake, Ciara Murphy, Caroline Donovan, Sonja March

**Affiliations:** 1 Australian Centre for Health Services Innovation (AusHSI) and Centre for Healthcare Transformation, School of Public Health & Social Work, Queensland University of Technology (QUT), Brisbane, Australia; 2 School of Applied Psychology, Griffith University, Brisbane, Australia; 3 School of Psychology & Counselling and Centre for Health Research, University of Southern Queensland, Springfield, Australia

**Keywords:** Mental health: model-based, Cost effectiveness, Economic evaluations, Child, Adolescents

## Abstract

Mental health disorders among children and youth are causing significant burden on health care systems. Hence, identifying cost-effective interventions is important for effective mental health care allocation. Although model-based economic evaluations are an essential component of assessing cost-effectiveness, evidence are limited in the context of child and youth mental health care. The objective was to systematically review the model-based economic evaluations of mental health interventions for children and youth.

**Methods**

Four databases (MEDLINE, EMBASE, PsycINFO and Web of Science) were searched using appropriate search terms to retrieve model-based economic evaluations of mental health interventions for children and youth. The reporting quality of the included studies were appraised using the Consolidated health economic evaluation reporting standards (CHEERS) checklist.

**Results**

The database search yielded 1921 records. Of the 12 selected for review, 66% were published after year 2015. Most of the studies were related to anxiety and post-traumatic stress disorder. There were eight cost-utility studies, three cost-effectiveness studies, and one study using both forms of analysis. Six studies used Markov models, three used decision trees, and three studies used both types of models. However, the model structure, health states, time horizon, and economic perspective showed wide variation. The reporting quality of the included studies varied from 91 to 96%.

**Conclusion**

Model based mental health economic evaluations among children and youth are increasingly being reported in recent research. The included studies used Markov models and decision trees, either alone or in combination, and the majority of the articles were of good reporting quality.

**Supplementary Information:**

The online version contains supplementary material available at 10.1186/s12913-022-07939-x.

## Background

Mental illness can impact a person’s cognitive, emotional and social abilities [1]. It is a growing cause of disability, with the last decade seeing a 13% rise in mental health conditions and substance use disorders [2]. According to the Global Burden of Disease Study 2019, disability-adjusted life years (DALYs) due to mental disorders were 125.3 million (95% CI 93.0–163.2), and the proportion of DALYs attributed to mental disorders were 4.9% (95% CI 3.9–6.1) [3]. Around 20% of the children and adolescents suffer from a mental health condition worldwide [2]; in the USA, 17.4% of the 2–8 years old children had a diagnosed mental, behavioural, or developmental disorder [4]. Australian Child and Adolescent Survey of Mental Health and Wellbeing 2013–2014 revealed that nearly 14% of Australian children aged 4 to 17 years experienced a mental disorder in the past 12 months [5].

Mental disorders are projected to become the leading cause of mortality and morbidity by 2030 [6]. This growth is extremely problematic given that mental health disorders are the single largest contributing source of global economic burden [7]. This is evidenced by the global expenditure on treating depression and anxiety rising to US$ one trillion per year [2]. Although there is a wide variety of evidence-based strategies that have been implemented to combat this growing issue, a reduction in the level of economic burden placed upon health systems and resources is yet to be experienced. In fact, the most discernible implementation barrier for mental health interventions is their often resource-intensive nature [7]. Not only does this place increased strain on health resource allocations, but it also makes treatment far less accessible for lower income families, as the cost often incurs out of pocket expenses [8, 9]. This issue becomes more evident given that the total cost of mental health problems and illness annually was 4000 Australian dollars (AUD) per person in Australia and 1400 Canadian dollars per person in Canada in 2016 [5, 10]. The annual additional population health care costs due to mental disorders among Australian children and adolescents is AUD$234, and of this, around 16% was attributed to out-of-pocket costs [11].

This is particularly problematic given that the types of interventions evidenced to result in the strongest improvements in mental health are those implemented earlier in life [12, 13]. The need for early intervention is even more apparent given that mental disorders are amongst the leading causes of disease burden in adolescents [14] with 13.9% of children and adolescents between 4 and 17 experiencing a mental health disorder [15]. Effective interventions, such as school based programmes, specialised mental health services, and community mental health care services designed to promote positive mental health in children and adolescents, have been shown to directly improve social and emotional skills and academic performance [12, 16]. Although the outcomes of such interventions are often reviewed positively, they often place great strain on resources. Early intervention is shown to have the strongest impact on reducing the prevalence and severity of mental illness throughout the lifespan, and therefore the need to investigate the potential of youth specific strategies is critical to reduce the overall economic burden of the disease [17]. As such, there has been increasing interest in the use of economic evaluations to determine the cost-effectiveness of mental health interventions and strategies. Such evaluations provide health planners with the ability to assess which interventions can provide the best value for money [18]. It is essential that intervention strategies are effective in reducing the burden of disease within the constraints of the allocated resources. Although economic evaluations can provide this information, it is important that the current scope of evaluations on youth-specific interventions is assessed in order to best inform future policy decisions.

Economic evaluations based on decision analytical models evaluate the cost effectiveness of available options, using information from different sources such as trials, meta-analyses, and observational studies [19]. Such evaluations can acknowledge a multitude of factors by integrating them into a single decision analytical framework [20], over a long period of time to capture differences in economic outcomes [19]. Hence, compared with single trial based economic evaluations, model based economic evaluations provide the best available evidence for decision makers [19]. Although model-based economic evaluations such as Markov models and discreet event simulations are an essential component of identifying cost-effective interventions [21], of the reviews to date, none have adequately assessed these health economic models concerning evaluating the cost-effectiveness of mental health interventions for children and youth. A recent systematic review of universal mental health interventions for children and adolescents identified only three model-based economic evaluations among the nine studies included [22]. However, this review was confined to providing an overview of the cost-effectiveness of different mental health interventions, rather than describing decision-analytic economic models and their methodological robustness in detail [22].

Furthermore, available evidence related to paediatric model-based economic evaluations is restricted to one geographic location. For example, a recent publication of the cost effectiveness of youth mental health interventions focused specifically on interventions implemented within the United States [7], Given these factors, there is a discernible gap in literature that provides an overview of model based economic evaluations for mental health interventions for children and youth, their methodological robustness and reporting quality. Hence, the objective of this study was to systematically review the model-based economic evaluations of mental health interventions for children and youth, to provide an overview of the decision analytic models utilised in these economic evaluations, assess their reporting quality, and provide guidance for future model based economic evaluations among children and youth. This review will provide information related to the structure and parameters of decision analytic models, which will be useful for future mental health related economic evaluations among children and youth.

## Methods

A systematic literature review was carried out to identify model-based economic evaluations of mental health interventions for children and youth, with the review protocol being registered with PROSPERO (registration number: CRD42021239391; https://www.crd.york.ac.uk/Prospero/). This systematic review followed the Preferred Reporting Items for Systematic Reviews and Meta-Analyses (PRISMA) guidelines for the systematic selection of articles [23]. The population, intervention, comparator, and outcome (PICO) for the review are as follows.

- Population: Child, adolescent or youth population (between 5 and 24 years)
- Intervention: Any non-pharmacological intervention, service use, or strategy for any mental health condition among children, adolescents or youth. The intervention could be either a preventive or a treatment (non-pharmacological) intervention.
- Comparator: Any control group or comparators assigned (no intervention or standard care) when comparing interventions, service use or strategy for any mental health condition among children, adolescents or youth
- Outcome: Any reported cost-effectiveness outcome in model based economic evaluations

Four databases (MEDLINE, EMBASE, PsycINFO and Web of Science) were searched for any model based economic evaluation of non-pharmacological interventions to improve the mental health of the children, adolescents and youth. Search terms were built around the words “Child/Youth/Adolescents”, “Mental health”, “Model based” and “Economic evaluations” with appropriate adjacency and truncation settings. Databases were searched until 11th December 2020 and no date restrictions were applied during the database search. Only English language articles were included in the review. The exact search terms are provided in Supplementary Fig. 1. After removing duplicates, search results were exported to the Rayyan QCRI, the systematic review web app (https://rayyan.qcri.org/reviews). The titles and the abstracts of the identified studies were reviewed by two independent reviewers (RH and CM) based on predefined inclusion and exclusion criteria. The articles eligible for full text reading were again reviewed by two independent reviewers (RH and SS) and conflicts were resolved by discussion with each other, and a third reviewer (SK).

**Fig. 1:**
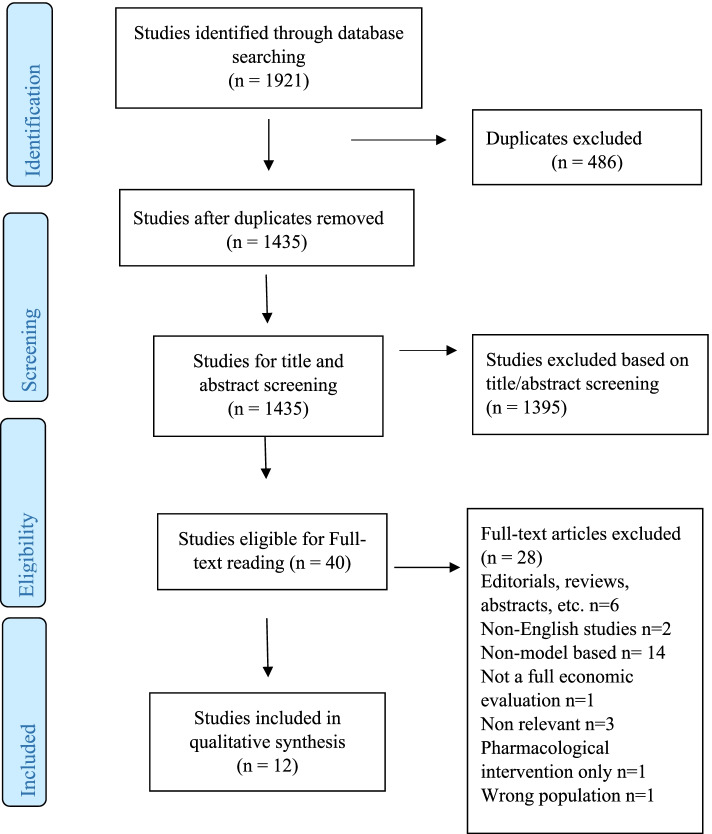
PRISMA Flow diagram

The articles were included if they were: based on either a child, adolescent or a youth population (between **5** and 24 years); non-pharmacological interventions for mental health; conducting a full economic evaluation which valued both costs and benefits of the intervention; an evaluation based on a decision-analytic model; a full publication or manuscript for review, and; written in English. The articles were excluded if: they were editorials, reviews, methods studies, letters or conference abstracts; the intervention was for any other disease where mental health promotion was a secondary outcome; only cost-analysis was performed; a comparator was not used; they were based only on adult population (i.e. interventions only for parents or/and teachers)**,** the economic evaluation was not based on a decision-analytic model. The process of the systematic selection of articles - including the number of records identified, screening for titles and abstracts, eligibility for full text reading, and papers included and excluded in the review - are outlined in the PRISMA flow diagram (Fig. 1). EndNote X8.2 (Thomson Reuters) was used as the reference manager.

The details of the studies which met inclusion criteria were recorded using data extraction tables by one researcher (RH), and the data for the 10% of included studies (two studies) were crosschecked by another independent reviewer (SS). The first summary table was used to record the basic study characteristics. The author and year, intervention or strategies being compared, country, study population, type of model, analytical method, assumptions, time horizon, discounting, main outcomes, sensitivity analyses, and other noteworthy features for each study were included in this table. For each comparator, incremental cost, incremental effectiveness/utility and incremental cost-effectiveness ratio(s) with uncertainty or confidence limits were recorded in the results table. When a study reported both trial based and model-based economic evaluations separately, only the data related to model-based economic evaluations were recorded. Markov models and decision trees are the most common modelling approaches used in health economic evaluations [19]. The Markov Model is a type of economic model that can model clinical problems with ongoing risk where recurrent events are essential to be considered. It assumes a patient is in one of a finite number of health states [24]. A decision tree summarises decisions and the probability or fraction of various outcomes hence it is more appropriate when recurring events are not important for the condition of interest and the timeframe is short [21].

Another summary data extraction table was used to assess the reporting quality of each study based on Consolidated Health Economic Evaluation Reporting Standards (CHEERS) checklist criteria [25]. The CHEERS checklist includes 24 item guidelines that should be included in economic evaluations publications. Two independent reviewers (RH and SS) assessed each selected article against each of the 24 CHEERS checklist items, with discrepancies resolved by discussion. Each CHEERS checklist item in the selected publication was scored as having met the criteria in full (“1”), not at all (“0”) or not applicable (NA). A score of “0” was also allocated if the item partially met the criteria, to avoid introducing subjectivity. The reporting quality of a study was expressed as a proportion of the items fully met for the article.

## Results

The database search yielded 1921 records of which1435 were screened for titles and abstracts after removing duplicates. A total of 1395 records were excluded based on titles and abstract reading. From the 40 studies eligible for full text reading, 28 met exclusion criteria, resulting in 12 articles being included in the final review (Fig. 1).

### Study characteristics

Of the 12 studies included, 66% were published after 2015. The studies were conducted mainly in Australia (*n* = 5) [26–30] and the UK (*n* = 3) [31–33] (Table 1). Most evaluations were related to anxiety and post-traumatic stress disorder (PTSD) (*n* = 5) [26, 27, 31, 32, 34], followed by depression (*n* = 3) [28, 29, 35] and anorexia nervosa (*n* = 2) [30, 36]. There were eight cost-utility studies, three cost-effectiveness studies, and one study with both cost utility and cost-effectiveness analyses. The majority of evaluations were from a healthcare system perspective (*n* = 8). Eleven studies discounted both costs and benefits, and one study [34] discounted costs only. The most commonly used discount rate was 3% (*n* = 6) (Table 1). Characteristics of the studies are provided in Table 2, while Table 3 describes the different model structures. For descriptive purposes, the studies were divided into four groups based on the disease condition of interest - anxiety and PTSD, depression, anorexia nervosa, and other conditions.

**Table 1:** General characteristics of the included studies (*n* = 12)

Characteristic	Number
**Year of publication**	
Before 2010	0
2010–2015	4
2016–2020	8
**Study country**	
Australia	5
UK	3
Sweden	1
The Netherlands	1
USA	1
UK and Ireland	1
**Mental health condition**	
PTSD & Anxiety	5
Depression	3
Anorexia nervosa	2
Other	2
**Health outcome**^**a**^	
QALY	6
DALY	4
Specific mental health outcome	4
**Study perspective**	
Health care system	8
Societal	1
Health care system and societal	1
Health care system and public payer	1
Not explicitly stated	1
**Discounting**	
3%	6
3.5%	3
Other	2
Justify why not discounted	1
**Type of economic model**	
Decision-tree	3
Markov model	6
Decision tree and Markov model	3

*DALY* Disability-adjusted life years, *QALY* Quality-adjusted life years

^a^Two studies evaluated more than one effectiveness measures

**Table 2:** Study characteristics

No	Authors, Year	Country	Study setting	Study population/ sample	Perspective	Intervention	Comparator	Discount rate
**Anxiety & PTSD**								
1	Simon E. et al., 2013 [34]	The Netherlands	School setting, then referral if necessary	Children (aged 8–12), high and median anxious	Societal	1. School-based screening child-focused intervention, 2. Screening and offering of a parent-focused intervention, 3. Screening and differentially offering a child- or parent focused intervention, based on parents’ anxious status	No intervention	Cost 4%
**2**	Gospodarevskaya E. & Segal L., 2012 [26]	Australia	Health care setting	10-year-old children who met either all or most of the PTSD diagnostic criteria	Health care system	1. Cognitive behavioral therapy (TF-CBT) 2. TF-CBT combined with selective serotonin reuptake inhibitor (SSRI) 3. Non-directive counselling	No treatment	Both cost and effects 5%
**3**	Mihalopoulos C. et al., 2015 [27]	Australia	Health care setting	Prevalent cases of PTSD estimated for the Australian population of 2012 children under 16 years	Health care system	Trauma-focused cognitive behavioural therapy (TF-CBT) in children	Current practice in Australia.	Both cost and effects 3%
**4**	Shearer J. et al., 2018 [31]	UK	Hospital setting	Children aged 8–17 years, diagnosed with PTSD	Health care system	Cognitive therapy for PTSD (CT-PTSD)	Usual care	Both cost and effects 3.5%
**5**	Mavranezouli I. et al., 2020 [32]	UK	National Health Service and personal social services in England	Children and young people (aged under 18 years) with clinically important PTSD symptoms	Health care system	10 psychological interventions 1. Cognitive therapy for PTSD (TF-CBT) 2. Cohen TF-CBT 3. Narrative exposure therapy (TF-CBT) 4. Exposure/ prolonged exposure therapy (TF-CBT) 5. Group CBT (TF-CBT) 6. Eye movement desensitisation and reprocessing (EMDR) 7. Family therapy 8. Play therapy 9. Parent training 10. Supportive counselling	No treatment	Both cost and effects 3.5%
**Depression**								
**6**	Mihalopoulos C. et al., 2012 [28]	Australia	School setting	11- to 17-year-old children and adolescents in the 2003 Australian population	Health care system	Population-level preventive intervention that screens children and adolescents for symptoms of depression in schools and the subsequent provision of a psychological intervention to those showing elevated signs of depression.	No intervention	Both cost and effects 3%
**7**	Lee YY. et al., 2017 [29]	Australia	Primary and secondary schools	Youth aged 11–17 years in the 2013 Australian population.	health and education sector	1) Universal prevention involving group-based psychological interventions delivered to all participating school students. 2) Indicated prevention involving group-based psychological interventions delivered to students with subthreshold depression.	No intervention	Both cost and effects 3%
**8**	Ssegonja R. et al., 2020 [35]	Sweden	School setting	A hypothetical homogeneous cohort of adolescents at a start-age of 15 years with subsyndromal depression (SD)	Health care system and limited societal perspective	Group based cognitive behavioural therapy (GB-CBT)	No intervention	Both cost and effects 3%
**Anorexia nervosa**								
**9**	Byford S. et al., 2019 [36]	UK and Ireland	Community-based secondary or tertiary child and adolescent mental health services (CAMHS)	Hypothetical cohort of children aged 8–17 years in contact with CAMHS for a first episode of anorexia nervosa	Health care system	Community-based specialist eating disorders services	Generic CAMHS care	Not done as the follow up not more than 12 months.
**10**	Le LK-D. et al., 2017 [30]	Australia	Youth aged 11–17 years in the 2013 Australian population.	The target population was 11–18 year olds with anorexia nervosa	Health care system	Family-based treatment (FBT) compared to adolescent-focused individual therapy (AFT)	No intervention	Both cost and effects 3%
**Other**								
**11**	Cottrell DJ. et al., 2018 [33]	UK	Child and Adolescent Mental Health Services (CAMHS) across three English regions.	Young people aged 11–17 years who had self-harmed at least twice presenting to CAMHS following self-harm.	Health care system	Family therapy (FT) delivered by trained and supervised family therapists (*n* = 415)	Treatment as usual (TAU) offered by local CAMHS following self-harm (*n* = 417)	Both cost and effects 3.5%
**12**	Freriks RD. et al., 2019 [37]	USA	Health care setting	Children 7–10 years, participated in Multimodal Treatment Study of Children With ADHD in United States	Not explicitly stated. But mentioned as societal cost and outcome in the abstract and discussion sections.	Three major forms of ADHD treatment (medication management, behavioral treatment, and the combination)	Routine community care	Both cost and effects 3%

**Table 3:** Comparison of model structures in the included studies

No	Authors, Year	Model Type	Time horizon	Model input parameters- Source	Model input parameters	Cost (Currency/ base year)	Effectiveness measure
**Anxiety & PTSD**							
**1**	Simon E. et al., 2013 [34]	Decision tree	2 years	Trial data	Cost, probabilities, children and parents scores for anxiety questionnaires (ADIS) to assess the presence and severity of anxiety diagnoses in the children)	2012 Euro	‘ADIS improved’ child
**2**	Gospodarevskaya E. & Segal L., 2012 [26]	Decision tree and Markov model	1 year decision tree 30 years Markov	Trial data, Existing databases and published literature	Clinical effectiveness for treating PTSD, probabilities for disease pathways, mortality, mental health care resource use, cost and utility values.	2010/2011 Australian dollars	Quality-adjusted life-years (QALYs) gained
**3**	Mihalopoulos C. et al., 2015 [27]*	Simulated population cohort decision-tree model	5 years	Existing databases and published literature	PTSD prevalence, proportions related to current practice of cognitive behavioral therapy (CBT), parameters related to benefits and resource use of CBT and utility values	2011/2012 Australian dollars	QALYs gained Disability-adjusted life-years (DALYs) averted
**4**	Shearer J. et al., 2018 [31]	Decision tree and Markov model	3 years (Decision tree- 11 weeks trial Markov 2 years 9 months)	Trial data and published literature	Intervention and comparator efficacy, transition probabilities, costs and utility values (By mapping parents’ Strengths and Difficulties Questionnaire scores to the Child Health Utility index- 9D using a published mapping algorithm	2014 British Pounds	QALYs gained
**5**	Mavranezouli I. et al., 2020 [32]	Decision tree and Markov model	3 years	Existing databases and published literature	Intervention cost per person for each psychological intervention utility data from Gospodarevskaya (2013) and Shearer et al. (2018)	2017 British Pounds	QALYs gained
**Depression**							
**6**	Mihalopoulos C. et al., 2012 [28]	Markov model	5 years	Existing databases and published literature	Proportions and efficacy data related to the intervention, duration and severity data related to depression, intervention costs, time and travel costs	2003 Australian dollars	DALYs averted
**7**	Lee YY. et al., 2017 [29]	Multiple cohort Markov model	10-year time horizon	Existing databases and published literature	Weighted average of global burden of disease 2013 disability weights for mild, moderate and severe depression Cost data and effect sizes	2013 Australian dollars	DALYs averted
**8**	Ssegonja R. et al., 2020 [35]	Markov cohort model	Two time horizons 1. 5 years 2. 10 years	Published literature	Intervention effectiveness inputs were derived from a recent systematic review and meta-analysis (Ssegonja et al., 2018). utility values Cost data	2018 US dollars	Proportion of cases of depression prevented QALYs gained
**Anorexia nervosa**							
**9**	Byford S. et al., 2019 [36]	Decision tree	12 months	Data collected in the cost study and followed the same timeline (12 months)	Probabilities -based on the number of people in each remission or relapse state in each arm at the two time points (i.e. The 6- and 12-month follow-ups) Costs estimated directly from the study data	2015/16 British pounds	Change in Children’s Global Assessment Scale score
**10**	Le LK-D. et al., 2017 [30]	Markov model	6 years	Published literature and existing databases	Intervention cost Disability weights Intervention and comparator efficacy, relapse and remission data	2013 Australian dollars	DALYs averted
**Other mental health conditions**							
**11**	Cottrell DJ. et al., 2018 [33]	Markov model	Up to 5 years after randomization.	Trial data and published literature	Participant cost and utility data were available at 6, 12 and 18 months from the trial data and were directly included into the model to estimate longer-term costs and health benefits	British pounds	QALY gained
**12**	Freriks RD. et al., 2019 [37]	Markov model	10 years beyond the 14-month trial period	Trial data- from a USA study, published literature and existing databases	Disease probabilities and treatment costs from trial data Criminal related cost-trial data- published literature and existing databases.	US dollars (Not mentioned)	Life-years of serious delinquent behavior prevented

*ADIS* Anxiety Disorder Interview Schedule, *CBT* cognitive behavioral therapy, *DALYs* Disability-adjusted life years, *PTSD* post traumatic stress disorder, *QALYs* Quality adjusted life years

*Details related to economic evaluation of children category is only reported here

### Anxiety and PTSD (*n* = 5)

Of the five studies related to anxiety and PTSD, two studies used only decision trees, while the remaining three used a combination of decision trees and Markov models as the decision analytic model (Table 3). The time horizon was less than 5 years in four studies. However, one study [26] modelled the impact for a longer time horizon by incorporating a decision tree for the first year and a Markov model for another 30 years and used a longer time horizon to capture long-term cost and outcomes for the model cohort. Improvement according to the Anxiety Disorder Interview Schedule (ADIS) Clinician Severity Rating scale [38] was the main effectiveness measure used for anxiety-related studies, whereas quality-adjusted life years (QALYs) was the main effectiveness measure for all four PTSD studies (Table 3). Model structure, health states, and the source of utility data showed wide variation among the included studies. The previously mentioned study using a 30-year time horizon [26], used nine PTSD related health states in the Markov model, while two other PTSD related studies [31, 32] used only two (PTSD and No PTSD) (Table 4). The utility values to calculate QALYs for the Markov health states were derived from published literature related to large-scale mental health surveys or within trials, using a generic preference-based quality of life measure (PBM). Assessment of Quality of Life (AQoL-4D) [26, 32] and the Child Health Utility index 9D (CHU-9D) [31, 32] were the generic PBMs used to derive utility values for PTSD studies (Table 4). Of the five anxiety and PTSD studies, one study [34] performed deterministic sensitivity analysis (DSA), two studies [27, 31] performed probabilistic sensitivity analysis (PSA), and two studies [26, 32] assessed parameter uncertainty using both DSA and PSA (Supplementary Table 1).

**Table 4:** Description of Markov model used in the included studies

No	Authors, Year	Health states used in the Markov model	Utility values
**Anxiety & PTSD**			
**1**	Gospodarevskaya E. & Segal L., 2012 [26]	Nine health states No PTSD/No depression PTSD only Depression only PTSD/Depression Death from suicide general population Death from suicide PTSD/depression Death from suicide depression Death from suicide PTSD Death from other causes	No PTSD/No depression (population norm) 10–30 year olds 0.87 30–40 year olds 0.85 PTSD only 0.61 (0.43–0.79) PTSD + depression 0.53 (0.37–0.69) Depression only 0.46 (0.32–0.60) (A paper from the first author with 2007 Australian National Survey of Mental Health and Wellbeing, Gospodarevskaya E., 2013. The 2007 Australian National Survey of Mental Health and Wellbeing collected data using generic preference-based instrument AQoL-4D).
**2**	Shearer J. et al., 2018 [31]	Two health states PTSD PTSD free	Not reported separately. Instead reported how the calculations were done for the trial arms. Utility values obtained by mapping parent-completed Strengths and Difficulties Questionnaire (SDQ) scores on to the Child Health Utility index 9D (CHU-9D) using a published mapping algorithm
**3**	Mavranezouli I. et al., 2020 [32]	Two health states PTSD No PTSD	Base-case analysis PTSD – 3-month 0.170 No-PTSD – 3-month 0.218 Secondary analysis PTSD – 3-month 0.185 No-PTSD – 3-month 0.193 Utility data from Gospodarevskaya (2013) and Shearer et al. (2018)
**Depression**			
**4**	Mihalopoulos C. et al., 2012 [28]	Two health states Depressed Non depressed	Not applicable as the outcome is DALYs averted
**5**	Lee YY. et al., 2017 [29]	Three health states Healthy Depression Dead	Not applicable as the outcome is DALYs averted
**6**	Ssegonja R. et al., 2020 [35]	Six health states Healthy Sub-syndromal depression Depressed Remission Recovered Dead	Utility values from published papers (Kolovos et al., 2017 [39], Burstrom et al., 2001 [40], and Burstrom et al., 2006 [41] - utility values based on EQ-5D Healthy 0.89 (0.78–0.95) (Burstrom et al., 2001, Burstrom et al., 2006) Subthreshold depression 0.62 (0.58–0.62) (Kolovos et al., 2017b) Depressed 0.39 (0.35–0.43) (Kolovos et al., 2017b; utility values based on EQ-5D) Remission 0.70 (0.67–0.73) (Kolovos et al., 2017b) Recovered 0.89 (0.78–0.95) (Burstrom et al., 2001, Burstrom et al., 2006) Dead 0.00 (0.00–0.00)
**Anorexia nervosa**			
7	Le LK-D. et al., 2017 [30]	Three health states People with anorexia Recovery Death	Not applicable as the outcome is DALYs averted
**Other mental health conditions**			
**8**	Cottrell DJ. et al., 2018 [33]	Three health states Self-harm (SH) No self-harm (noSH) Death	Utility values obtained using EQ-5D within the study. Health state utilities in treatment as usual arm 6 months- SH 0.760 (SE 0.161) 12 months- SH 0.751 (SE 0.187) noSH 0.784 (SE 0.180) Death 0 18 months- SH 0.754 (SE 0.033) noSH 0.808 (SE 0.157) Death 0 Health state utilities in family therapy arm 6 months- SH 0.799 (SE 0.178) 12 months- SH 0.793 (SE 0.184) noSH 0.813 (SE 0.194) Death 0 18 months- SH 0.732 (SE 0.239) noSH 0.823 (SE 0.179) Death 0
**9**	Freriks RD. et al., 2019 [37]	Three health states No delinquency Minor to moderate delinquency Serious delinquency	Not applicable as the outcome is life-years of serious delinquent behavior prevented

*DALYs* Disability-adjusted life years, *PTSD* post traumatic stress disorder, *QALYs* Quality adjusted life years

### Depression (*n* = 3)

All three studies related to depression used Markov models, and the time horizons were less than 10 years (Table 3). Two studies [28, 29] used disability adjusted life years (DALYs) averted as the main effectiveness measure, and the model had three health states (healthy/non-depressed, disease/depressed, and dead) in their Markov model. The other study [35] used QALYs gained as the main effectiveness measure and included six health states (Healthy, sub-syndromal depression, depressed, remission, recovered, and dead) in the Markov model (Table 4). This study derived the utility values for depression health states from published literature based on EuroQoL-5D (EQ-5D) utility values (Table 4). All three depression studies [28, 29, 35] used both DSA and PSA approaches to assess parameter uncertainty (Supplementary Table 1).

### Anorexia nervosa (*n* = 2)

Of the two studiers related to Anorexia Nervosa, one study [36] used a decision tree and modelled only the trial data for 1 year. The other study [30] used a Markov model, based on published literature and existing databases, and the time horizon was 6 years (Table 3). The Markov model of both the studies had three health states (people with anorexia nervosa, recovery, and death), and the main effectiveness measure was DALYs averted. These two studies [30, 36] performed both DSA and PSA as the sensitivity analysis (Supplementary Table 1).

### Other mental health conditions/ strategies (*n* = 2)

Two studies assessed the cost-effectiveness of mental health conditions/ strategies other than anxiety, PTSD, depression or anorexia nervosa. Of them, one study assessed the cost-effectiveness of a self-harm prevention intervention [33] using a Markov model with three health states (self-harm, no self-harm, and death). The main effectiveness measure of the study was QALYs gained, and the utility values were derived within the trial using EQ-5D questionnaire [33] (Table 4). Another study assessed the cost-effectiveness of an intervention for attention-deficit hyperactivity disorder (ADHD) using a Markov model. The model used three delinquency related health states (no delinquency, mild to moderate delinquency, and serious delinquency) and the main effect measure was life-years (LYs) of serious delinquent behaviour prevented (Table 3). Of note was the inclusion of serious delinquency state as the absorbing health state for the Markov model in this study [37]. Of the two studies in this group, one [37] performed DSA, whereas the other [33] performed both DSA and PSA to assess the parameter uncertainty (Supplementary Table 1).

### Reporting quality of selected articles

The evaluation of each included article against the CHEERS checklist criteria is provided in Supplementary Table 2. The reporting quality varied from 91 to 96%. All included studies reported the choice of model, model assumptions, and analytic methods in detail. Furthermore, all included studies reported study parameters, incremental costs and outcomes, and results related to the characterising uncertainty as text, table or figures in their results section. Altough all studies reported the methods and resources for the estimation of cost data, only 83% provided a full description of the currency, price date, and conversion. The CHEERS item complied with the least among included studies, was the characterising heterogeneity (item 21).

## Discussion

This paper summarises the model based economic evaluations of mental health interventions among children and youth. The review identified 12 studies published until 2020 December. However, all included papers were published after 2010, indicating that model based economic evaluations of children and youth mental health interventions have attracted attention in only recent times. The included studies used Markov models, decision trees or a combination of these methods as the decision analytic approach. However, the model structure, health states, time horizon and perspective demonstrated wide variation. The reporting quality of the included studies was more than 90% based on CHEERS criteria.

The structure of the model based economic evaluations depends on the target disease and the research question [19]. Markov models and decision trees either alone or in combination, are the most common modelling approaches used in health economic evaluations [19], and was evident in our review as well. Mental disorders are chronic in nature and have long-term impact with periods of recovery and relapse [42]. Compared with decision trees, Markov models allow modelling of the cost-effectiveness of an intervention using recurring health states over a longer time horizon [19]. Most of the included studies have used Markov models either alone or in combination with decision trees, making them more appropriate for chronic conditions such as mental health disorders. However, the model structures, health states and economic perspectives used in the included studies showed wide variation, even within similar mental health conditions. For example, among the three studies using Markov models to evaluate interventions for PTSD, one study used a complex model structure with nine health states [26], whereas two others used only two health states [31, 32]. Five studies have used death as the absorbing health state in the Markov model. The wide variation in model structures, time frames, and economic perspectives, even within similar health conditions, has previously been reported in systematic reviews of model-based economic evaluations in other areas of health research as well [39]. The perspective of the model-based mental health economic evaluations would play a vital role in assessing its usefulness for decision making. However, more than 60% of the studies have used only the health system perspective for the model inputs. The difficulty of obtaining real-time cost data related to the cost incurred due to the broad societal impact of mental health problems would be the reason for this variation. Nevertheless, it is a noteworthy that few studies have tried to incorporate the cost data related to societal perspective such as loss of productivity. One of the included studies [29] related to a school based mental health intervention had incooperated the cost to the education system along with the health system cost. Another study [37], though did not explicitly mention the study perspective, have used cost to criminal justice system along with the health care system cost. Such studies would have provided more information and aided policy decisions on implementing the interventions in real-world settings.

Using model parameters from relevant and valid sources is important for an economic evaluation that produces evidence for policymakers to use with confidence [40]. All included studies in this review gathered model input parameters from either existing valid database, published literature, or trial data. Although mental health disorders have long term impact, most studies have used shorter time horizons, with 11 studies having time horizons 10 years or less. This is likely due to the consideration of intervention impact during childhood only, or due to features related to the specific mental health condition. For example, three PTSD studies included in the current review used time horizons less than 5 years, indicating that those suffering with PTSD may recover within 3 years. In contrast, one study related to PTSD used a longer time horizon (30 years) to capture the long-term cost effectiveness of the intervention. However, the authors reported that they made a number of assumptions due to limited availability of evidence over a long period of time. Mental health conditions are chronic conditions that are associated with long term social and economic impacts. A limited time horizon therefore impacts the accuracy of analysis [42]. From this review, it is evident that it is a challenge to gather valid model input parameters over a longer period for model based, mental health intervention economic evaluations. Future research into mental health interventions for children and youth should allow for a longer follow-up duration to ensure robust evidence for policy makers.

This review found that where utility values were derived for Markov models to calculate QALYs as the main outcome measure, they were generated from large scale mental health surveys or within trial analysis. The utility values obtained from large scale mental health surveys provide valid and reliable data, and therefore their continued use is encouraged in future model based mental health intervention economic evaluations. However, it is advisable to consider whether the method of deriving utility values through published literature is appropriate or relevant to the study population of interest, prior to applying these values in future evaluations. The utility values in large-scale mental health surveys or within trial analysis in this review were derived using generic preference-based quality of life measures (PBM), mainly the EQ-5D [41] and the AQoL-4D questionnaires. The EQ-5D is the most widely used generic PBM to generate utility values in many areas of health, as well as being the recommended tool by many health technology assessment agencies [43]. However, only one study [31] in this review used utility values generated from a paediatric generic PBM, CHU-9D [44], with another study [32] using CHU-9D utility values only for secondary analyses. From this review it is clear that although the EQ-5D has a pediatric version -EQ-5D youth [45] - it has not been the chosen tool for generating utility values when evaluating mental health interventions for children and youth. Limited use of pediatric PBM to derive utility values may be due to the unavailability of pediatric-specific value sets or utility values for mental health states using children and youth. PBM specifically designed for children and youth would have been more sensitive to capturing the effectiveness of mental health interventions targeting pediatric populations. Therefore, we recommend widely available pediatric PBM tools when generating utility values for mental health states among children and youth in future research. All studies included in this present review performed some form of sensitivity analysis, which is useful in assessing the parameter uncertainty. Compared to the deterministic sensitivity analysis approach, probabilistic sensitivity analysis would give a more complete picture of impact of parameter uncertainty [40] and is therefore considered to be the recommended approach for examining robustness of the model.

### Strengths and limitations

One of the strengths of this review is that all necessary steps have been taken to ensure all relevant studies were included in the review. We selected databases that are known for their relevance in the field of medical and mental health research as well as health economics, and these databases were searched systematically with search terms that were built using appropriate wordings, with necessary adjacency and truncation settings to identify all relevant articles. Another strength of this review is the screening of eligible articles by two independent researchers to reduce potential bias, with articles screened using validated methods and tools. Although a potential limitation of this study was the inclusion of only English-language articles (Which may have impacted the overall number of articles included), there appeared to be only a limited number of articles identified from non-English speaking countries. Additionally, due to wide variations in the model structure, health states, time horizon and economic perspective used, even within the same mental health condition, direct quantitative comparison across all mental health interventions was not possible.

#### Implication of findings

This paper summarised the model-based economic evaluations of mental health interventions among children and youth. It provided information about the structure and parameters of decision-analytic models available in the literature. Hence, findings from this review would be useful for future mental health-related economic evaluations among children and youth. Mental health problems are long-term and pose a considerable impact on society beyond health. However, as discussed in detail, minimal data are available for the cost incurred due to the societal impact of the mental health problems, and it is a challenge to gather valid model input parameters over a more extended period. Availability of such data would aid researchers to showcase the more significant impact of their intervention. Hence, these factors should be considered, and maximum effect should be taken to incorporate data related to a broader perspective when planning the economic evaluation of mental health interventions.

## Conclusion

In conclusion, the present review identified 12 model-based economic evaluations of mental health interventions among children and youth. All included articles were published after 2010, indicating that this is an area of research that is becoming increasingly evaluated and reported. The included studies have used Markov models and decision trees, either alone or in combination. However, it was implausible to pool the data due to wide variations in the model structure, health states, time horizon and economic perspective. The majority of the articles were of good reporting quality based on CHEERS checklist criteria, although provision of the details related to currency, price date, and conversion and characterising heterogeneity are areas to be improved up.

## Supplementary Information

**Additional file 1: Supplementary Figure 1.** Search Terms. **Supplementary Table 1.** Reporting of cost effectiveness analysis. **Supplementary Table 2.** Assessment of reporting quality of included studies using CHEERS checklist.

## Data Availability

Relevant data on the details of the included studies are provided in the supplementary materials.

## Abbreviations

ADHD: Attention-deficit hyperactivity disorder
ADIS: Anxiety Disorder Interview Schedule
AQoL-4D: Assessment of Quality of Life
AUD: Australian dollars
CHEERS: Consolidated Health Economic Evaluation Reporting Standards checklist
CHU-9D: Child Health Utility index 9D
DALYs: Disability adjusted life years
DSA: Deterministic sensitivity analysis
EQ-5D: EuroQoL-5D
LYs: Life-years
PBM: Preference-based quality of life measure
PICO: Population, intervention, comparator, and outcome
PRISMA: Preferred Reporting Items for Systematic Reviews and Meta-Analyses guidelines
PSA: Probabilistic sensitivity analysis
PTSD: Post-traumatic stress disorder
QALY: Quality-adjusted life years
