# Supplementary material for: Cost-effectiveness analysis of paediatric mental health interventions: a systematic review of model-based economic evaluations
Source: BMC Health Serv Res. 2022 Apr 22;22:542. doi: 10.1186/s12913-022-07939-x (PMC9034631; doi:10.1186/s12913-022-07939-x)
Supplement: Supplementary file 1 — Additional file 1: Supplementary Figure 1. Search Terms. Supplementary Table 1. Reporting of cost effectiveness analysis. Supplementary Table 2. Assessment of reporting quality of included studies using CHEERS checklist. [file 12913_2022_7939_MOESM1_ESM.docx]

**Cost-effectiveness analysis of paediatric mental health interventions: A systematic review of model-based economic evaluations**

**Running title: Mental health economic models for Children/ youth**

**Supplementary Figure 1: Search Terms**

1# (child) OR (children) OR (adolescen*) OR (adolescents) OR (adolescent*) OR ('school age*') OR ('school age') OR ('school based') OR (youth*) OR (young) OR (juvenile) OR (kid*) OR (minor) OR (teen) OR (teenagers) OR ('young adult') OR (underage*)

2# (anxiety) OR (depression) OR (mental* well-being*) OR '(psychologica*l well-being*) OR (mental* health*) OR (mental* ill*) OR (mental* disorder*) OR (mental* diseas*) OR (mental* AND state*) OR (depress*) OR (anxiet*) OR (anxious) OR (hypervigilance) OR (nervous*) OR (emotion*) OR (stress) OR (internalizing) OR MH(anxiety) OR MH(depression)

3# (Cost benefit analysis) OR (cost effective*) OR (cost-effective*) OR (cost utility) OR (cost-utility) OR (cost benefit) OR (cost-benefit) OR (quality-adjusted life years) OR (health economic) OR MH(economic evaluation)

4# (markov model) OR (discrete event simulation) OR (decision tree) OR (decision-analytic model)

1# AND 2# AND 3# AND 4#

**Supplementary Table 1: Reporting of cost effectiveness analysis**

| **No** | **Authors, Year** | **Incremental cost** | **Incremental utility/ incremental effect** | **ICER** | **WTP threshold** | **Sensitivity analysis** | **Conclusion** |
| --- | --- | --- | --- | --- | --- | --- | --- |
|  | **Anxiety & PTSD** | |  |  |  |  |  |
| **1** | Simon E. et al., 2013 (1) | - Strategy 1 (Child-focused intervention): €1311.10 - Strategy 2 (Parent-focused intervention): €1,304.01 - Strategy 3 (Screening and a child- or parent focused intervention): €1,297.06 - Strategy 4 (Do nothing): €1,295.99 | Strategy 1: 0.19  Strategy 2: 0.18  Strategy 3: 0.19  Strategy 4: 0.18 | Incremental costs per extra ‘Anxiety Disorder Interview Schedule scale- clinician severity rating’ improved child €107 (Strategy 3 versus 4) | €3,000 extra per improved child | Scenario analysis  Explorative one-way sensitivity analyses by increasing and decreasing cost and probabilities by 25%. Results were fairly robust. | “Screening, followed by offering the parent focused intervention to children of anxious parents, and the child-focused intervention to children of non-anxious parents had the highest incremental effects at low incremental costs compared to do nothing’’ (1) |
| **2** | Gospodarevskaya E. & Segal L., 2012 (2) | Compared to ‘no treatment’:   - Non-directive counselling:   A$ 2123.20   - Cognitive behavioral therapy (TF-CBT) only:   A$ 2095.7   - TF-CBT + selective serotonin reuptake inhibitor (SSRI):   A$ 2269.8 | Compared to ‘no treatment’:   - Non-directive counselling: 1.02 - TF-CBT only: 1.28 - TF-CBT + SSRI: 1.34 | Compared to ‘no treatment’:   - Non-directive counselling: 2081.57 - TF-CBT only: 1650.16 - TF-CBT + SSRI: 1706.61 | A$ 50,000 per QALY | Deterministic and probabilistic sensitivity analyses. All model parameters other than unit costs and population utility norms.  Deterministic:   - 30% variation range used when no evidence available for measure of variability   Probabilistic :   - Beta or Gamma probability distribution were assigned where appropriate and the Monte-Carlo simulation was used.   Results were robust for most of the parameter variations. | “Even after accounting for uncertainty in parameter estimates, the results of the modelled economic evaluation demonstrated that all psychotherapy treatments for PTSD in sexually abused children have a favourable ICER relative to no treatment” (2) |
| **3** | Mihalopoulos C. et al., 2015 (3) | Compared to current practice  0.87 $Millions (0.28–2.2 95% UI) | QALY: 0.09 (95% UI 0.00 to0.25) DALY: 0.10 (95% UI 0.00–0.27) | TF-CBT is highly cost-effective compared to current practice at $8900/QALY (95% UI 5200–28,000), $8000/DALY (95% UI 4600–25,000) in children. | $50,000/  QALY or DALY value-for-money threshold | Probabilistic sensitivity analysis- binomial, uniform or log normal probability distributions were assigned where appropriate and the Monte Carlo simulation modelling was used.  99% of iterations fell below the WTP value. | “The three Guideline recommended interventions (2 adults and 1 child) evaluated in this study are likely to have a positive impact on the economic efficiency of the treatment of PTSD if adopted in full“ (3) |
| **4** | Shearer J. et al., 2018 (4) | At 3 years compared to the usual care, CT-PTSD £97 | At 3 years compared to the usual care,  CT-PTSD 0.0577 | ICER at 3 years was £2,205 per QALY | UK £20,000 to £30,000 per QALY | Probabilistic sensitivity analysis – Beta and Gamma probability distribution were assigned where appropriate.  Results showed CT-PTSD had a probability of being cost-effective compared to usual care of between 69% and 75% at the £20,000 -£30,000/ QALY threshold. | “This study provides preliminary evidence for the cost-effectiveness of cognitive therapy in this treatment population. Larger pragmatic trials with longer follow-up are indicated” (4) |
| **5** | Mavranezouli I. et al., 2020 (5) | Incremental cost and effectiveness for each intervention compared to the no treatment were presented as a figure with cost-effectiveness plane | | Presented as a cost-effectiveness plane | £20,000/QALY | One-way deterministic sensitivity analyses to explore the impact of a change in the annual risk of relapse (varied between zero and 0.20). Results were overall robust to assumptions tested.  Probabilistic sensitivity analysis – Beta, Gamma and normal probability distribution were assigned where appropriate. Cost-effectiveness acceptability frontier has been plotted with 10,000 iterations.  Four scenario based probabilistic analysis -performed with utility data from different literature sources.  results were varied based on the scenario. | “Individual forms of TF-CBT and, to a lesser degree, play therapy appear to be cost-effective in the treatment of children and young people with PTSD. Family therapy and supportive counselling are unlikely to be cost-effective relative to other interventions” (5) |
|  | **Depression** |  |  |  |  |  |  |
| **6** | Mihalopoulos C. et al., 2012 (6) | Total costs  Median (95% uncertainty interval)  31 $Millions (13 $Millions to 58 $Millions) | DALY averted Median (95% uncertainty interval)  5600 (1000 to 11000) | Median (95% uncertainty interval)  $5400 ($1400 to $32000) per DALY averted | $50 000 per DALY | Multivariate probabilistic and univariate sensitivity analysis.  Probabilistic- using Monte Carlo simulation modelling and Beta, triangle, Poisson, normal, binomial and discrete distributions were applied where appropriate. Results were robust to model assumptions.  Univariate- assessed based on scenarios. 2% of iterations falling above a $50 000 per DALY value-for-money threshold. | “Screening children for signs of depression and the provision of a psychological intervention to prevent a diagnosable case of MDD represents very good value for money” (6) |
| **7** | Lee YY. et al., 2017 (7) | Net costs (95% uncertainty interval  AU$ thousands   - Universal psychological 21802 (−75 to 55 743) - Indicated psychological 58843 (23460 to 102 573) | Face to face interventions DALY averted (95% uncertainty interval)   - Universal psychological 3367 (1618–5184) - Indicated psychological 4083 (1295–9361) | Mean (95% UI)   - Universal psychological $7350 (Dominant to 23070) per DALY averted - Indicated psychological $19 550 (3081 to 56 713) per DALY averted | $50 000 per DALY averted | Probabilistic and univariate sensitivity analysis  Uncertainty analyses – using Monte Carlo simulation and Lognormal and Pert distributions for parameters were applied where appropriate. Baseline ICERS were generally robust to changes in model assumptions.  Univariate sensitivity analyses- to assess the effect of changing various parameters (mainly to 50% and 100%). internet-delivered interventions were highly cost-effective when assuming intervention effect sizes of 100 and 50% relative to face-to-face delivered interventions. | “School-based psychological interventions appear to be cost-effective. However, realizing efficiency gains in the population is ultimately dependent on ensuring successful system-level implementation” (7) |
| **8** | Ssegonja R. et al., 2020 (8) | Incremental cost mean (95% UI)  5 years:  -313 (-2,141 to -207)  10 years:  -349 (-2,858 to -31) | Incremental QALY mean (95% UI)  5 years: 0.12  (-0.36 to 0.57)  10 years: 0.15  (-0.79 to1.05)  Incremental cases prevented mean (95% UI)  5 years: 0.07 (0 to 0.14)  10 years: 0.10  (-0.05 to 0.25) | GB-CBT is dominant | $ 20000/ QALY | Uncertainty analyses – using Monte Carlo simulation and varying parameters with different time horizons. Probability distributions were not reported. Cost effectiveness planes were presented. Intervention demonstrated a probability of being cost-effective of over 95%.  Univariate (one-way)- by changing the key parameters in different variation ranges. In sensitivity analysis results were robust to the modelling assumptions. | “GB-CBT indicated preventive interventions for depression in adolescents seem to have a potential to be good value for money” (8) |
| Anorexia nervosa | | |  |  |  |  |  |
| **9** | Byford S. et al., 2019 (9) | Incremental analysis was not performed, and authors stated that it was not appropriate for the economic modelling because there was no comparison between the two groups; instead, the model explored the total cost per gain in outcome for the full population of young people, dependent on the proportion of those young people who are initially assessed in specialist services or generic CAMHS | | | Presented as a figure based on percentage of young persons assessed by the specialist eating disorder services. | One-way sensitivity analysis and probabilistic sensitivity analysis. Sensitivity analyses suggesting that cost per 10-point improvement in CGAS score varies little as the percentage of participants taking the specialist or generic pathway is varied. | “Decision modelling did not support the hypothesis that changes to the provision of specialist services would generate savings for the NHS, with results and sensitivity analyses suggesting that cost per 10-point improvement in CGAS score varies little as the percentage of participants taking the specialist or generic pathway is varied” (9) |
| **10** | Le LK-D. et al., 2017 (10) | Total costs Mean (95% UI)   - Family-based treatment (FBT) $10,600,559 ($8,798,924 to $12,527,236) - Adolescent-focused individual therapy (AFT) $13,383,861 ($11,225,374 to $15,700,089) - No intervention $9,878,893 ($8,481,095 to $11,293,603) | DALYs averted (95% UI)   - FBT versus AFT 74 (26–130) - FBT versus no intervention   142 (74–225)   - AFT versus no intervention   65 (2–146) | - FBT versus AFT Dominant (Dominant to Dominant) - FBT versus no intervention $5,089 (Dominant to $16,659) - AFT versus no intervention $51,897 ($21,591 to $1,712,491) | $50,000 per DALY averted | Multivariate probabilistic and series of one-way and two-way sensitivity analyses  Probabilistic- using Monte Carlo simulation and Lognormal, Gamma, Beta and Pert distributions for parameters were applied where appropriate.  FBT and AFT are 100% and 45% likely to be cost-effective at AUD $50,000 per DALY averted.  One way and two way-  With different variation ranges  indicated that excluding hospital costs led to increases in the ICERs but the conclusion of the study did not change. | “FBT is the most cost-effective among treatment arms, whereas AFT was not cost effective compared to no intervention” (10) |
| Other | | |  |  |  |  |  |
| **11** | Cottrell DJ. et al., 2018 (11) | Family therapy (FT) compared to Treatment as usual (TAU)  Mean (95% confidence interval-CI) £1262.13 (1106.62 to 1417.62) | FT compared to TAU  5-year QALY gain Mean (95% CI)  0.065 (0.053 to 0.075) | £19,486.97 per QALY | £20,000–30,000 per QALY | Probabilistic sensitivity analyses- using Monte Carlo simulation and Beta, fixed and Lognormal, distributions for parameters were applied where appropriate.  Long term cost effectiveness plane and cost effectiveness acceptability curves presented- high level of uncertainty around the cost-effectiveness of FT compared with TAU over a 5-year horizon.  Sensitivity analysis-  Series of one-way Deterministic sensitivity analysis was performed based on different scenarios- Results depends on the scenarios. | “FT was unlikely to be cost-effective in most sensitivity analyses and was dominated by TAU in the complete-case analysis” (11) |
| **12** | Freriks RD. et al., 2019 (12) | - Mean discounted total cost presented.   Medication management $1779  Behavioral treatment $9170  Combined treatment $10527  Routine community care $1537 | - The discounted average years of serious delinquent behavior prevented were presented   Medication management 7.86  Behavioral treatment 7.90 Combined treatment 8.17  Routine community care 8.10 | ICER not presented.  Net-monetary benefit (NMB) reported.  Mean NMB  Medication management $95,449  Behavioral treatment $88,553  Combined treatment $90,536  Routine community care $98,660 | $12,370 (Annual cost associated with serious delinquency in children with ADHD was taken as WTP value) | Deterministic sensitivity analyses with linearly increasing WTP thresholds between $0 and $50,000- Estimates remained stable after linearly increasing the WTP threshold between $0 and $50,000 | “Three major forms of ADHD treatment turned out to be inferior to the control condition” (12) |

*ADHD- Attention-Deficit/Hyperactivity Disorder; AFT- adolescent-focused individual therapy; ARMS-At-risk mental state; CAMHS- Generic child and adolescent mental health services; CT-PTSD: Cognitive therapy for PTSD; FBT- Family-based treatment; FT- Family Therapy; GB-CBT- Group based cognitive behavioural therapy; ICER- Incremental cost effectiveness ratio; NMB- Net Monetary Benefit; PTSD-Post traumatic stress disorder; QALYs- Quality-adjusted life years; SSRI- Selective serotonin reuptake inhibitor; TAU- Treatment as usual; TFCBT- Cognitive behavioral therapy; 95% UI- 95% Uncertainty interval; WTP- Willingness to pay*

**Supplementary Table 2: Assessment of reporting quality of included studies using CHEERS checklist**

|  |  | **Study** | **Simon E. et al., 2013** | **Gospodarevskaya E. & Segal L., 2012** | **Mihalopoulos C. et al., 2015** | **Shearer J. et al., 2018** | **Mavranezouli I. et al., 2020 2020** | **Mihalopoulos C. et al., 2012** | **Lee YY. et al., 2017** | **Ssegonja R. et al., 2020** | **Byford S. et al., 2019** | **Le LK-D. et al., 2017** | **Cottrell DJ. et al., 2018** | **Freriks RD. et al., 2019** |
| --- | --- | --- | --- | --- | --- | --- | --- | --- | --- | --- | --- | --- | --- | --- |
| Title & Abstract | 1 | **Title** | 1 | 1 | 1 | 1 | 1 | 1 | 1 | 1 | 1 | 1 | NA | 1 |
|  | 2 | **Abstract** | 1 | 1 | 1 | 1 | 1 | 1 | 1 | 1 | 1 | 1 | 1 | 1 |
| Introduction | 3 | **Background and objectives** | 1 | 1 | 1 | 1 | 1 | 1 | 1 | 1 | 1 | 1 | 1 | 1 |
| Methods | 4 | **Target population and subgroups** | 1 | 1 | 1 | 1 | 1 | 1 | 1 | 1 | 1 | 1 | 1 | 1 |
|  | 5 | **Setting and location** | 1 | 1 | 1 | 1 | 1 | 1 | 1 | 1 | 1 | 1 | 1 | 1 |
|  | 6 | **Study perspective** | 1 | 1 | 1 | 1 | 1 | 1 | 1 | 1 | 1 | 1 | 1 | 0 |
|  | 7 | **Comparators** | 1 | 1 | 1 | 1 | 1 | 1 | 1 | 1 | 1 | 1 | 1 | 1 |
|  | 8 | **Time horizon** | 1 | 1 | 1 | 1 | 1 | 1 | 1 | 1 | 1 | 1 | 1 | 1 |
|  | 9 | **Discount rate** | 1 | 1 | 1 | 1 | 1 | 1 | 1 | 1 | 1 | 1 | 1 | 1 |
|  | 10 | **Choice of health outcomes** | 1 | 1 | 1 | 1 | 1 | 1 | 1 | 1 | 1 | 1 | 1 | 1 |
|  | 11b | **Measurement of effectiveness** | 1 | 1 | 1 | 1 | 1 | 1 | 1 | 1 | 1 | 1 | 1 | 1 |
|  | 12 | **Measurement and valuation of preference-based outcomes** | NA | 1 | 1 | 1 | 1 | 1 | 1 | 1 | NA | 1 | 1 | NA |
|  | 13b | **Estimating resources and costs** | 1 | 1 | 1 | 1 | 1 | 1 | 1 | 1 | 1 | 1 | 1 | 1 |
|  | 14 | **Currency, price date, and conversion** | 1 | 1 | 1 | 1 | 1 | 1 | 1 | 1 | 1 | 1 | 0 | 0 |
|  | 15 | **Choice of model** | 1 | 1 | 1 | 1 | 1 | 1 | 1 | 1 | 1 | 1 | 1 | 1 |
|  | 16 | **Assumptions** | 1 | 1 | 1 | 1 | 1 | 1 | 1 | 1 | 1 | 1 | 1 | 1 |
|  | 17 | **Analytic methods** | 1 | 1 | 1 | 1 | 1 | 1 | 1 | 1 | 1 | 1 | 1 | 1 |
| Results | 18 | **Study parameters** | 1 | 1 | 1 | 1 | 1 | 1 | 1 | 1 | 1 | 1 | 1 | 1 |
|  | 19 | **Incremental costs and outcomes** | 1 | 1 | 1 | 1 | 1 | 1 | 1 | 1 | NA | 1 | 1 | 1 |
|  | 20b | **Characterizing uncertainty** | 1 | 1 | 1 | 1 | 1 | 1 | 1 | 1 | 1 | 1 | 1 | 1 |
|  | 21 | **Characterizing heterogeneity** | 0 | 0 | 0 | 0 | 0 | 0 | 0 | 0 | 0 | 0 | 0 | 1 |
| Discussion | 22 | **Study findings, limitations, generalizability, and current knowledge** | 1 | 1 | 1 | 1 | 1 | 1 | 1 | 1 | 1 | 1 | 1 | 1 |
| Other | 23 | **Source of funding** | 1 | 1 | 1 | 1 | 1 | 1 | 1 | 1 | 1 | 1 | 1 | 1 |
|  | 24 | **Conflicts of interest** | 1 | 1 | 1 | 1 | 1 | 1 | 1 | 1 | 1 | 1 | 1 | 1 |
|  |  |  | **0.91*** | **0.96** | **0.96** | **0.96** | **0.96** | **0.96** | **0.96** | **0.96** | **0.95*** | **0.96** | **0.91*** | **0.91*** |

**Percentage calculated as number complied with the number of applicable criteria.*

**References**

1. Simon E, Dirksen CD, Bögels SM. An explorative cost-effectiveness analysis of school-based screening for child anxiety using a decision analytic model. European Child and Adolescent Psychiatry. 2013;22(10):619-30.

2. Gospodarevskaya E, Segal L. Cost-utility analysis of different treatments for post-traumatic stress disorder in sexually abused children. Child and Adolescent Psychiatry and Mental Health. 2012;6.

3. Mihalopoulos C, Magnus A, Lal A, Dell L, Forbes D, Phelps A. Is implementation of the 2013 Australian treatment guidelines for posttraumatic stress disorder cost-effective compared to current practice? A cost-utility analysis using QALYs and DALYs. Australian and New Zealand Journal of Psychiatry. 2015;49(4):360-76.

4. Shearer J, Papanikolaou N, Meiser-Stedman R, McKinnon A, Dalgleish T, Smith P, et al. Cost-effectiveness of cognitive therapy as an early intervention for post-traumatic stress disorder in children and adolescents: a trial based evaluation and model. Journal of child psychology and psychiatry, and allied disciplines. 2018;59(7):773-80.

5. Mavranezouli I, Megnin-Viggars O, Trickey D, Meiser-Stedman R, Daly C, Dias S, et al. Cost-effectiveness of psychological interventions for children and young people with post-traumatic stress disorder. Journal of child psychology and psychiatry, and allied disciplines. 2020;61(6):699-710.

6. Mihalopoulos C, Vos T, Pirkis J, Carter R. The population cost-effectiveness of interventions designed to prevent childhood depression. Pediatrics. 2012;129(3):e723-e30.

7. Lee YY, Barendregt JJ, Stockings EA, Ferrari AJ, Whiteford HA, Patton GA, et al. The population cost-effectiveness of delivering universal and indicated school-based interventions to prevent the onset of major depression among youth in Australia. Epidemiology and Psychiatric Sciences. 2017;26(5):545-64.

8. Ssegonja R, Sampaio F, Alaie I, Philipson A, Hagberg L, Murray K, et al. Cost-effectiveness of an indicated preventive intervention for depression in adolescents: a model to support decision making. Journal of Affective Disorders. 2020;277:789-99.

9. Byford S, Petkova H, Stuart R, Nicholls D, Simic M, Ford T, et al. Alternative community-based models of care for young people with anorexia nervosa: the CostED national surveillance study. Health Services and Delivery Research 2019;7(37).

10. Le LK-D, Barendregt JJ, Hay P, Sawyer SM, Hughes EK, Mihalopoulos C. The modeled cost-effectiveness of family-based and adolescent-focused treatment for anorexia nervosa. The International journal of eating disorders. 2017;50(12):1356-66.

11. Cottrell DJ, Wright-Hughes A, Collinson M, Boston P, Eisler I, Fortune S, et al. A pragmatic randomised controlled trial and economic evaluation of family therapy versus treatment as usual for young people seen after second or subsequent episodes of self-harm: the Self-Harm Intervention - Family Therapy (SHIFT) trial. Health technology assessment (Winchester, England). 2018;22(12):1-222.

12. Freriks RD, Mierau JO, van der Schans J, Groenman AP, Hoekstra PJ, Postma MJ, et al. Cost-Effectiveness of Treatments in Children With Attention-Deficit/Hyperactivity Disorder: A Continuous-Time Markov Modeling Approach. MDM policy & practice. 2019;4(2):2381468319867629.
